# Multifractal based topological characterization of perovskite crystal and predictive analysis on its physical properties

**DOI:** 10.3389/fchem.2025.1639522

**Published:** 2025-08-04

**Authors:** K. Yogalakshmi, D. Easwaramoorthy

**Affiliations:** Department of Mathematics, School of Advanced Sciences, Vellore Institute of Technology, Vellore, Tamil Nadu, India

**Keywords:** fractal analysis, generalized fractal dimensions, topological indices, edge partition technique, reverse degree, *CaTiO*_*3*_ perovskite crystal

## Abstract

Perovskite material has gained popularity and attracted much attention in recent years. Calcium Titanium Oxide $\left(CaTiO_{3}\right)$ is a perovskite crystal structure, which has the molecular formula $ABX_{3}$ , widely employed in geosciences, electronic ceramic materials, and radioactive waste immobilization. The powerful mathematical tool called topological descriptors or index is used to analyze $CaTiO_{3}$ perovskite. It provides a numerical representation of certain physical and chemical features on the crystal structure. Additionally, topological indices are frequently used by chemical scientists to determine the strain energy, melting and boiling points, distortion, and stability of chemical substances. In this paper, the topological indices are derived from the M-polynomial for $CaTiO_{3}$ perovskite crystal structure depends on reverse degree using the edge partitioning technique and the behavior of this crystal structure is presented as numerically derived equations and topological descriptors. The Generalized Fractal Dimensions (GFD) are calculated using Renyi entropy based on the equations of the reverse degree dependent indices derived from the M-polynomial, which are compared graphically and tabularly with respect to given indices. Furthermore, the molecular mass and collision diameter physical properties of the $CaTiO_{3}$ crystal structure are analyzed and correlated with the generalized fractal dimensions derived from topological indices, leading to the development of a predictive correlation model explaining the highest and lowest correlation coefficient values of each index defined in this phenomenon. Therefore, GFD aids to understand how the fractal nature, connectivity, and branching of the crystal affect its properties in the growth pattern of the $CaTiO_{3}$ crystal structure.

## 1 Introduction

The fractal geometry is a form of mathematical geometry with irregular design that studies complex, self-similar structures and repeating patterns at various scales. In contrast to conventional Euclidean geometry, which works with smooth forms like lines and circles, fractal geometry works with objects that are frequently asymmetrical and broken, but nonetheless show some sort of structure and order. Furthermore, Benoit Mandelbrot formally defined the concept of fractals in 1975, establishing that a fractal is a set whose Hausdorff dimension strictly surpasses its topological dimension. Mandelbrot was also the pioneer in introducing the fractal geometry. The term “fractal”originates from the Latin word “fractus”, which translates to fractured or broken (Addison, 1997; Falconer, 2003; Mandelbrot, 1982). Fractals are typically generated through recursive formulas or iterative processes, where simple mathematical principles are applied repeatedly to develop intricate patterns. Moreover, these objects retain their appearance regardless of the level of magnification, whether zoomed in or out (Barnsley, 1993).

The fractal geometry is particularly useful for analyzing and estimating the intricate properties of natural objects, offering a more nuanced approach than traditional geometric methods. Also, the fractal geometry continues to be a vibrant and growing field that intersects with natural phenomena, computer graphics, art, signal and image processing, science, and engineering. Its exploration has enhanced our comprehension of the complexity inherent present in both mathematical concepts and natural systems. The fractal dimension is a non-integer single-valued, standard measure used to describe fractal structures (Banerjee et al., 2021; Edger, 2013). The fractal dimension is insufficient in the analysis of complex structures used with the fractal concept. Therefore, such an irregular complex structures are characterized using Generalized Fractal Dimensions (GFD) or Renyi Fractal Dimensions. These generalized dimensions are also employed to analyze, classify, and assess complex crystal networks. In this paper, the perovskite crystal structure is examined using GFD method (Thangaraj and Easwaramoorthy, 2022; Uthayakumar and Easwaramoorthy, 2013; Yogalakshmi and Easwaramoorthy, 2024; Yogalakshmi and Easwaramoorthy, 2025).

One of the minerals, Calcium Titanium Oxide or Calcium Titanate $\left(CaTiO_{3}\right)$ is the basis for the term “perovskites,” which was named after its discovery by the Russian mineralogist Count Lev Aleksevich Von Perovski. The class of materials known as perovskites, with the general formula $ABX_{3}$ , displays a range of outstanding physicochemical properties. These include ferroelectricity, ionic conductivity, dielectric characteristics, superconductivity, and the ability to undergo an insulator-metal transition. In perovskites, the two cations, A and B, differ in size and are bonded with the anion X, which is typically an oxygen atom. The cations in group A are generally alkaline earth metals (such as Ca, Mg, Sr, etc.) and are slightly larger than the cations in group B, which are usually transition metals (Garcia-Mendoza et al., 2023; Ng et al., 2021). $CaTiO_{3}$ exhibits different phases at varying temperatures. When synthesized at temperatures below 1107° C, it adopts an orthorhombic structure. As the temperature rises to 1227° C, it transitions to a tetragonal phase. Above 1300° C, $CaTiO_{3}$ transforms into a cubic phase with the space group $Pm\bar{3}m$ . The cubic form of $CaTiO_{3}$ perovskite is particularly well-suited for various device applications, including capacitors, piezoelectric devices, transducers, displays, wireless communications, random access memories, tunable microwave components, and sensors (Ali and Yashima, 2005; Rizwan et al., 2020).

Of these, titanium oxide $\left(TiO_{2}\right)$ naturally exists in three different crystallographic forms: rutile, brookite, and anatase. Anatase $TiO_{2}$ is the most commonly employed photocatalyst for the oxidative degradation of organic compounds and has been thoroughly studied due to its significant effectiveness in photocatalysis and photon-electron transfer (Weon et al., 2018). Calcium oxide (CaO) , which crystallizes in a cubic system, is a highly valuable industrial compound. It is used as a catalyst, in the treatment of toxic waste, as an additive in refractory materials, in paints, and in various other essential applications. Ultrafine metal oxide particles can be utilized as bactericides and adsorbents. Calcium oxide has also demonstrated significant potential as an effective adsorbent for neutralizing toxic chemical agents (Ramola et al., 2019). The properties of $CaTiO_{3}$ type perovskites are significantly affected by factors such as lattice defects at the A or B cation sites, the material’s nature, particle size, morphology, and the exposed surface area. These factors are frequently investigated using Density Functional Theory (DFT), which offers valuable physical insights into various phenomena and also acts as a crucial tool for predicting the properties of unexplored materials (Rizwan et al., 2020).

Mathematical chemistry is a field that applies mathematical techniques to address challenges in chemistry. A key aspect of this field is chemical graph theory, which focuses on the topological properties of molecular structures, including the mathematical analysis of isomerism and the creation of topological indices or descriptors (Liu et al., 2019; Trinajstic, 1992; Arockiaraj et al., 2024). Topological indices, which remain unchanged under graph isomorphism, are employed to predict a range of features of chemical compounds and nanomaterials, such as viscosity, radius of gyration, and boiling point. Cheminformatics is a growing field that employs QSPR (Quantitative Structure-Property Relationships) and QSAR (Quantitative Structure-Activity Relationships) to assess the biological activity and features of nanomaterials. This method predicts the behavior of chemical compounds by utilizing topological indices and physico-chemical characteristics (Ashraf and Idrees, 2024; Khabyah et al., 2023; Peter et al., 2025; Zuo et al., 2022; Daniel et al., 2025).

Polynomials also have applications in chemistry, similar to topological indices. For instance, the Hosoya polynomial (or Wiener polynomial) is crucial for calculating distance based topological indices. Similarly, the M-polynomial, introduced in 2015, is important for computing various degree based topological indices. The primary benefit of the M-polynomial is the detailed information it provides about degree based graph invariants (Afzal et al., 2020; Chaudhry et al., 2021; Liu et al., 2019; Zuo et al., 2022). Also, first and second reverse Zagreb polynomials and first and second reverse hyper-Zagreb polynomials have been presented to the crystallographic structure of molecules using the reverse degree (Wang et al., 2020).

Dongming Zhao et al. determined reverse degree-based topological indices for polycyclic metal organic networks, specifically TM-TCNB (Zhao et al., 2022). Likewise, Ali Khabyah et al. calculated certain descriptors for two-dimensional coronene fractal structures using reverse degree-based topological indices (Khabyah et al., 2023). In another study, Ali N.A. Koam et al. analyzed various reverse degree-based indices for the sodalite materials network. They also explored the structural behavior of this network, discussed related topological descriptors, and developed numerical equations to model it Koam et al. (2022). Xuewu Zuo et al. have computed topological indices for the molecular structure of polyphenylene using M-polynomials (Zuo et al., 2022). Faryal Chaudhry et al. derived the M-polynomial and topological indices for the tadpole graph and subsequently used the M-polynomial to retrieve numerous topological indices (Chaudhry et al., 2021).

Perovskite materials like calcium titanium oxide $\left(CaTiO_{3}\right)$ play a vital role in various emerging technologies, such as optoelectronics applications, photovoltaics, and catalysis processes. However, quantitative structure-property correlation is a challenge because of their intricate 3D atomic arrangements. It analyzes and comprehends these intricate systems using strong, mathematically based methods. This research presents a novel contribution, as reverse degree-based topological indices via M-polynomial and GFD analysis have not yet been explored for the selected $CaTiO_{3}$ structure. In this new study, the 3D $CaTiO_{3}$ perovskite nanostructure is characterized using GFD analysis in combination with Renyi entropy, reverse topological indices, and M-polynomial. A novel method for measuring structural complexity and heterogeneity in crystalline materials is provided by this combined approach.

The framework of this study is presented as follows. Section 2 covering the preliminaries required for this research. Section 3 describes the methodology for this study. In Section 4, the general formulae for reverse degree based topological indices using M-polynomials to assess the properties of $CaTiO_{3}$ crystal material are derived. Section 5 presents the GFD results, and concludes with the correlation model and its application to examine the relationship between the measured GFD and potential physical properties in Section 6. In Section 7, concluding remarks are finally provided.

## 2 Preliminaries

This section discusses reverse degree based topological indices, M-polynomial, and generalized fractal dimensions derived from Renyi entropy.

### 2.1 Reverse degree based topological descriptors

A molecular graph is a basic connected graph in the field of Chemical Graph Theory (CGT), whose atoms are regarded as vertices and bonds as edges. Throughout this work, we will refer to G as the simple connected graph, $E_{G}$ as the edge set, and $V_{G}$ as the vertex set. Vertex degree is represented by $d_{\tau }$ , which is the number of vertices that are connected to the vertex τ (Peter et al., 2025). Researcher Kulli (2018) developed an additional concept known as reverse degree and represented by the symbol of Rd . The mathematical expression for this recently developed idea is $Rd_{\tau }=1+\Delta -d_{\tau }$ , where Δ is the greatest degree in a graph. An edge partition of a graph, denoted by the label $E_{Rd_{\tau },Rd_{\upsilon }}$ , is based on the idea of the reverse-degree of the end vertices of an edge $e=\tau \upsilon \in E_{G}$ . Likewise, the notion $\left|E_{Rd_{\tau },Rd_{\upsilon }}\right|$ offered as its cardinality (Wang et al., 2020; Koam et al., 2022).

In (Zhao et al., 2022; Khabyah et al., 2023), the authors developed a topological descriptor or index (RTI(G)) as a reverse degree based mapping from the vertex set of $G\;\left(V_{G}\right)$ to the set of all real numbers, mentioned below.

$$RTI\left(G\right)=\underset{e\in E_{G}}{\sum }\omega \left(Rd_{\tau },Rd_{\upsilon }\right),$$

$$where\omega \left(Rd_{\tau },Rd_{\upsilon }\right)={\left(\frac{Rd_{\tau }*Rd_{\upsilon }}{Rd_{\tau }+Rd_{\upsilon }-2}\right)}^{3}.$$

Then, RTI(G) emerged as a version of the augmented Zagreb index RAZI(G) in reverse degree. Moreover, Table 1 provides the reverse degree formulae for a range of topological indices.

**TABLE 1:** Reverse degree dependent topological indices derived from M-polynomial of a graph G .

Indices	Reverse degree based formula	Derivation from M[G;x,y]
First Zagreb Index	$RM_{1}\left(G\right)=\sum_{e\in E_{G}}Rd_{\tau }+Rd_{\upsilon }$	$\left(D_{x}+D_{y}\right)\left(M\left[G;x,y\right]\right){|}_{x=y=1}$
Second Zagreb Index	$RM_{2}\left(G\right)=\sum_{e\in E_{G}}Rd_{\tau }*Rd_{\upsilon }$	$\left(D_{x}D_{y}\right)\left(M\left[G;x,y\right]\right){|}_{x=y=1}$
Modified Second Zagreb Index	$R^{m}M_{2}\left(G\right)=\sum_{e\in E_{G}}\frac{1}{Rd_{\tau }*Rd_{\upsilon }}$	$\left(S_{x}S_{y}\right)\left(M\left[G;x,y\right]\right){|}_{x=y=1}$
General Randic Index	$RR_{\alpha }\left(G\right)=\sum_{e\in E_{G}}{\left(Rd_{\tau }*Rd_{\upsilon }\right)}^{\alpha }$	$\left(D_{x}^{\alpha }D_{y}^{\alpha }\right)\left(M\left[G;x,y\right]\right){|}_{x=y=1}$
Forgotten Index	$RFI\left(G\right)=\sum_{e\in E_{G}}\left(Rd_{\tau }^{2}+Rd_{\upsilon }^{2}\right)$	$\left(D_{x}^{2}+D_{y}^{2}\right)\left(M\left[G;x,y\right]\right){|}_{x=y=1}$
Harmonic Index	$RH\left(G\right)=\sum_{e\in E_{G}}\frac{2}{Rd_{\tau }+Rd_{\upsilon }}$	$2S_{x}J\left(M\left[G;x,y\right]\right){|}_{x=1}$
Symmetric Degree Division Index	$RSDD\left(G\right)=\sum_{e\in E_{G}}\frac{Rd_{\tau }^{2}+Rd_{\upsilon }^{2}}{Rd_{\tau }*Rd_{\upsilon }}$	$\left(D_{x}S_{y}+S_{x}D_{y}\right)\left(M\left[G;x,y\right]\right){|}_{x=y=1}$
Inverse Sum Indeg Index	$RISI\left(G\right)=\sum_{e\in E_{G}}\frac{Rd_{\tau }*Rd_{\upsilon }}{Rd_{\tau }+Rd_{\upsilon }}$	$S_{x}JD_{x}D_{y}\left(M\left[G;x,y\right]\right){|}_{x=1}$
Augmented Zagreb Index	$RAZI\left(G\right)=\sum_{e\in E_{G}}{\left(\frac{Rd_{\tau }*Rd_{\upsilon }}{Rd_{\tau }+Rd_{\upsilon }-2}\right)}^{3}$	$S_{x}^{3}Q_{-2}JD_{x}^{3}D_{y}^{3}\left(M\left[G;x,y\right]\right){|}_{x=1}$

### 2.2 M-polynomial

A graph polynomial is a mathematical tool associated with a graph that generally characterizes the graph in a manner that is invariant under graph isomorphism. In the past, numerous algebraic graph polynomials have been created that can also describe the properties of molecular structures. A robust method for generating topological indices involves in computing the graph polynomial and then using integrals or derivatives of the polynomial at specific points to obtain the topological descriptor. This process is known as the Hosoya polynomial method (Zuo et al., 2022). The M-polynomial functions, similarly to the Hosoya polynomial, yields expressions related to topological indices that depend on vertex degrees. It is considered one of the most effective graph polynomials developed to date. This suggests that by analyzing the M-polynomial for the molecular structure, we can typically derive a closed formula for certain specific indices. For a graph G , the M-polynomial defined in Liu et al. (2019), Chaudhry et al. (2021) expressed as:

$$M\left[G;x,y\right]=\underset{\delta \leq i\leq j\leq \Delta }{\sum }m\left(i,j\right)\left[G\right]x^{i}y^{j}.$$ (1)

where $\delta =Min\left\{d_{\tau }:\tau \in V_{G}\right\}$ , $\Delta =Max\left\{d_{\tau }:\tau \in V_{G}\right\}$ and m(i,j)[G] is the number of edges $\tau \upsilon \in E_{G}$ such that $\left\{d_{\tau },d_{\upsilon }\right\}=\left\{i,j\right\}$ .

### 2.3 Renyi entropy based GFD measurements

When calculating Generalized Fractal Dimensions (GFD), Renyi entropy offers a potent framework for describing the multiscale complexity and diverse spatial distributions present in molecular structures. A probability distribution’s diversity or uncertainty is measured in information theory using the Renyi entropy, which is a generalization of the Shannon entropy. It was first presented by Alfred Renyi in 1961, and a non-negative real number q serves as its parameter. Shannon entropy quantifies the mean amount of uncertainty in a system, whereas Renyi entropy adds a parameter that, depending on its value, permits a wider range of measurements. By changing the parameter q , Renyi entropy provides a flexible method of measuring uncertainty. Moreover, employing the Renyi entropy parameter q allows for focused analysis of specific regions within the probability distribution, facilitating a thorough examination of structural variations in $CaTiO_{3}$ systems.

The following defines the Renyi entropy of order q for a discrete probability distribution $\left\{p_{1},p_{2},\ldots ,p_{N}\right\},p_{i}\in \left[0,1\right],\forall i\in \left\{1,2,\ldots ,N\right\}$ (Easwaramoorthy and Uthayakumar, 2011; Uthayakumar and Easwaramoorthy, 2012; Uthayakumar and Easwaramoorthy, 2013):

$$R_{q}=\frac{1}{1-q}ln\left(\overset{N}{\underset{i=1}{\sum }}p_{i}^{q}\right).$$ (2)

where q is a real number that is non-negative and does not equal 1. q=1 is a specific case, where the Renyi entropy deduces to the Shannon entropy.

Based on Equation 2, the Generalized Fractal Dimensions or Renyi Fractal Dimensions, of order q∈(−∞,∞) (excluding q=1 ) for well-defined probability distributions can be expressed as follows (Easwaramoorthy and Uthayakumar, 2011; Uthayakumar and Easwaramoorthy, 2012; Uthayakumar and Easwaramoorthy, 2013; Yogalakshmi and Easwaramoorthy, 2024; Thangaraj and Easwaramoorthy, 2022).

$$D_{q}=\lim_{\varepsilon \to 0}\frac{1}{q-1}\frac{ln\left(\sum_{i=1}^{N}p_{i}^{q}\right)}{ln\varepsilon }.$$ (3)

Here, GFD signifies the Generalized Renyi Entropy.

## 3 Methodology

### 3.1 Reverse degree based topological descriptors via M-Polynomial

The M-polynomial is a generating function that provides a unified structure for deriving different topological indices and encodes information about a molecular graph based on reverse degrees. The reverse degrees of the vertices connected by each edge are used to define the M-polynomial. It is constructed by grouping edges based on the reverse degrees of their endpoints and then summing the contributions from each of these edge classes.

This study investigates the three-dimensional structure of $CaTiO_{3}$ , a component of the crystal network, based on various reverse degree dependent topological indices, as indicated in Table 1. Furthermore, over the last 5 years (Afzal et al., 2020), the M-polynomial of numerous chemical graphs has been defined. In the present study, we developed M-polynomial of the $CaTiO_{3}$ perovskite crystal structure. This graph polynomial allowed us to compute a number of reverse degree dependent topological indices, which are presented in Table 1. A few well-known reverse degree dependent topological indices that were calculated using the M-polynomial are listed in Table 1. The following are definition of various operators used in Table 1:

$$\begin{array}{cc} D_{x}=x\frac{\partial \left(f\left(x,y\right)\right)}{\partial x}, & D_{y}=y\frac{\partial \left(f\left(x,y\right)\right)}{\partial y},S_{x}=\int_{0}^{x}\frac{f\left(t,y\right)}{t}dt,S_{y}=\int_{0}^{y}\frac{f\left(x,t\right)}{t}dt,\\ & J\left(f\left(x,y\right)\right)=f\left(x,x\right),andQ_{\alpha }\left(f\left(x,y\right)\right)=x^{\alpha }f\left(x,y\right). \end{array}$$

where M[G;x,y] is denoted by the symbol f(x,y) .

The edge partition method separates the total set of edges into multiple groups based on the reverse degrees of their endpoint vertices, allowing for the calculation of indices related to reverse degrees. The partitioning is defined as follows:

$$Rm\left(i,j\right)\left[G\right]=\left|\left\{\tau \upsilon \in E_{G}:\left(Rd_{\tau },Rd_{\upsilon }\right)=\left(i,j\right),\forall i,j\geq 1\right\}\right|.$$

The symbol η is defined as the $sup_{\tau \in V_{G}}\left\{Rd_{\tau }\right\}$ , where $Rd_{\tau }$ denotes the reverse degree of vertex τ in $V_{G}$ . Based on this, we introduce the following category:

$$K=\left\{\left(i,j\right)\in N\times N:1\leq i\leq j\leq \eta \right\}.$$

Using edge partitions based on reverse degrees, the M-polynomial defined in Equation 1 can be reformulated as follows:

$$M\left[G;x,y\right]=\underset{\delta \leq i\leq j\leq \Delta }{\sum }Rm\left(i,j\right)\left[G\right]x^{i}y^{j}.$$ (4)

where $\delta =Min\left\{Rd_{\tau }:\tau \in V_{G}\right\}$ , $\Delta =Max\left\{Rd_{\tau }:\tau \in V_{G}\right\}$ .

For instance, when considering the reverse degree based first and second Zagreb indices derived from the M-polynomial,

$$\begin{array}{cc} RM_{1}\left(G\right) & =\left(x\frac{\partial }{\partial x}+y\frac{\partial }{\partial y}\right)\left(M\left[G;x,y\right]\right){|}_{x=y=1}.\\ RM_{2}\left(G\right) & =\left(x\frac{\partial }{\partial x}.y\frac{\partial }{\partial y}\right)\left(M\left[G;x,y\right]\right){|}_{x=y=1}. \end{array}$$

### 3.2 Proposed GFD for self-similar $CaTiO_{3}$ perovskite crystal

In this section, multifractal analysis is discussed to understand the complexity of the $CaTiO_{3}$ perovskite crystal structure based on reverse degree based topological indices derived from the M-polynomial using generalized fractal dimensions.

The following approach is employed to introduce the probability distribution using the general formula for reverse degree based topological indices via the M-polynomial.

Let N denote the number of unique self-similar crystallographic orbits in the perovskite crystal structure, and let ε represent the scaling factor of this structure. In this way, the probability function for an edge (e) used in the generalized fractal dimensions interpretation with Renyi entropy is adjusted to $p_{e}$ . This function is defined for all edges of G through the reverse topological index via the M-polynomial using Equation 4, and is employed to estimate the Generalized Fractal Dimensions (GFD) of the $CaTiO_{3}$ perovskite crystal structure G .

$$p_{e}=\frac{Rm\left(i,j\right)\left[G\right]x^{i}y^{j}}{M\left[G;x,y\right]},wherexandyare\mathrm{fi}xedrealnumberande\in E_{G}.$$

Therefore, from Equations 3, 4, the formula for the generalized fractal dimensions, which is based on the reverse degree dependent topological indices via M-polynomial represented by $D_{q}^{RTI}\left(M\left[G;x,y\right]\right)$ , can be introduced as follows:

$$D_{q}^{RTI}\left(M\left[G;x,y\right]\right)=\lim_{\varepsilon \to 0}\frac{1}{q-1}\frac{ln\left(\sum_{e\in E_{G}}p_{e}^{q}\right)}{ln\varepsilon }.$$ (5)

Using Equation 5, the reverse degree and M-polynomial based GFD can be modified as follows:

$$D_{q}^{RTI}\left(M\left[G;x,y\right]\right)=\lim_{\varepsilon \to 0}\frac{1}{q-1}\frac{ln\left(\sum_{e\in E_{G}}{\left(\frac{Rm\left(i,j\right)\left[G\right]x^{i}y^{j}}{M\left[G;x,y\right]}\right)}^{q}\right)}{ln\varepsilon }.$$ (6)

## 4 Main results on topological indices for $CaTiO_{3}$

**FIGURE 1:**
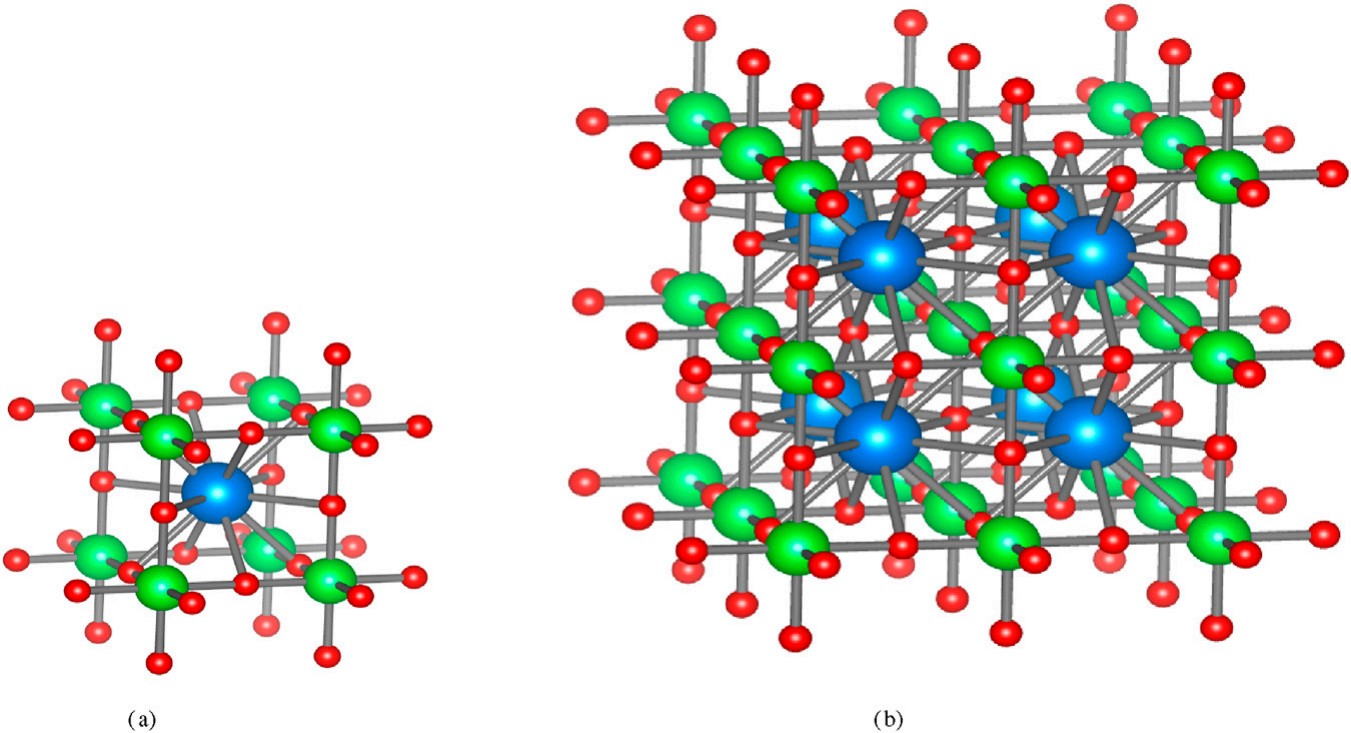
Perovskite crystal structure of $CaTiO_{3}$ (calcium titanium oxide). **(a)** $\left[CaTiO_{3}\left(1,1,1\right)\right]$ . **(b)** $\left[CaTiO_{3}\left(2,2,2\right)\right]$ .

In this section, we provide generalized formulae for calculating reverse degree-based topological indices using the M-polynomial of the $CaTiO_{3}$ perovskite crystal through the edge partition technique. Further we expand the method for obtaining the complex structure information through GFD measures. In Figure 1a, the unit cell of the $CaTiO_{3}$ perovskite crystal contains 45 vertices and 60 edges. Within this structure, blue atoms represent “Ca”, green atoms denote “Ti”, and red atoms indicate “O”. As shown in Figure 1b, the unit cell is extended to multiple layers in r×s×t arrangement. This resulting structure is referred to as $CaTiO_{3}\left(r,s,t\right)$ .

We determined that $CaTiO_{3}\left(r,s,t\right)$ comprises 6(r+s+t)+5(rs+st+rt+rst)+7 vertices and 6(r+s+t+rs+st+rt+3rst+1) edges. Additionally, the partition of $CaTiO_{3}$ reveals three distinct types of edges based on the degrees of the end vertices in $CaTiO_{3}\left(r,s,t\right)$ . These are detailed in Table 2. The studied edge partition quantities and Equation 1 could be used to derive equations for the reverse degree-based topological indices through from the M-polynomial. Theorem 1, Theorem 2 can be proved by using the information in Tables 1, 2.

**TABLE 2:** Reverse degree based edge partition of $CaTiO_{3}\left(r,s,t\right)$

$\left(d_{\tau },d_{\upsilon }\right)$	$\left(Rd_{\tau },Rd_{\upsilon }\right)$	$\left|E_{Rd_{\tau },Rd_{\upsilon }}\right|$
(3,12)	(1,10)	12rst
(3,6)	(7,10)	3(r+1)(s+1)(t+1)
(1,6)	(7,12)	3(r+1)(s+1)(t+1)

Theorem 1 *The M-polynomial of* $CaTiO_{3}\left(r,s,t\right)$ *;* r,s,t≥1 *is* $M\left[CaTiO_{3}\left(r,s,t\right);x,y\right]=12rstxy^{10}+3\left(r+1\right)\left(s+1\right)\left(t+1\right)x^{7}y^{10}+3\left(r+1\right)\left(s+1\right)\left(t+1\right)x^{7}y^{12}.$

Proof. From Figure 1, the edge set of $CaTiO_{3}\left(r,s,t\right)$ is divided into the following three parts:

$$E_{1,10}=\left\{e=\tau \upsilon \in E\left(CaTiO_{3}\left(r,s,t\right)\right)|Rd_{\tau }=1,Rd_{\upsilon }=10\right\},$$

$$E_{7,10}=\left\{e=\tau \upsilon \in E\left(CaTiO_{3}\left(r,s,t\right)\right)|Rd_{\tau }=7,Rd_{\upsilon }=10\right\},$$

$$E_{7,12}=\left\{e=\tau \upsilon \in E\left(CaTiO_{3}\left(r,s,t\right)\right)|Rd_{\tau }=7,Rd_{\upsilon }=12\right\},$$

such that

$$|E_{1,10}|=12rst,\;|E_{7,10}|=3\left(r+1\right)\left(s+1\right)\left(t+1\right),\;|E_{7,12}|=3\left(r+1\right)\left(s+1\right)\left(t+1\right).$$

Now

$$\begin{array}{cc} M\left[CaTiO_{3}\left(r,s,t\right);x,y\right] & =\underset{\delta \leq i\leq j\leq \Delta }{\sum }m\left(i,j\right)\left[CaTiO_{3}\left(r,s,t\right)\right]x^{i}y^{j},\\ & =\underset{1\leq i\leq j\leq 12}{\sum }m\left(i,j\right)\left[CaTiO_{3}\left(r,s,t\right)\right]x^{i}y^{j},\\ & =\underset{1\leq 10}{\sum }m\left(1,10\right)\left[CaTiO_{3}\left(r,s,t\right)\right]xy^{10}+\underset{7\leq 10}{\sum }m\left(7,10\right)\left[CaTiO_{3}\left(r,s,t\right)\right]x^{7}y^{10}\\ & \;+\underset{7\leq 12}{\sum }m\left(7,12\right)\left[CaTiO_{3}\left(r,s,t\right)\right]x^{7}y^{12},\\ & =|E_{1,10}|xy^{10}+|E_{7,10}|x^{7}y^{10}+|E_{7,12}|x^{7}y^{12},\\ & =12rstxy^{10}+3\left(r+1\right)\left(s+1\right)\left(t+1\right)x^{7}y^{10}+3\left(r+1\right)\left(s+1\right)\left(t+1\right)x^{7}y^{12}. \end{array}$$

Theorem 2 *Let* $CaTiO_{3}\left(r,s,t\right)$ *;* r,s,t≥1 *be a calcium titanium oxide perovskite crystal structure. Then,*

1. $RM_{1}\left[CaTiO_{3}\right]=240rst+108\left(rs+rt+st+r+s+t+1\right)$ ,
2. $RM_{2}\left[CaTiO_{3}\right]=582rst+462\left(rs+rt+st+r+s+t+1\right)$ ,
3. $R^{m}M_{2}\left[CaTiO_{3}\right]=1.27857rst+0.07857\left(rs+rt+st+r+s+t+1\right)$ ,
4. $RR_{\alpha }\left[CaTiO_{3}\right]=\left(12\times 10^{\alpha }+3\times 7^{\alpha }\left(10^{\alpha }+12^{\alpha }\right)\right)rst+\left(3\times 7^{\alpha }\left(10^{\alpha }+12^{\alpha }\right)\right)\left(rs+rt+st+r+s+t+1\right)$ ,
5. $RFI\left[CaTiO_{3}\right]=2238rst+1026\left(rs+rt+st+r+s+t+1\right)$ ,
6. $RH\left[CaTiO_{3}\right]=2.85055rst+0.66873\left(rs+rt+st+r+s+t+1\right)$ ,
7. $RSDD\left[CaTiO_{3}\right]=134.47857rst+13.27857\left(rs+rt+st+r+s+t+1\right)$ ,
8. $RISI\left[CaTiO_{3}\right]=36.52518rst+25.61609\left(rs+rt+st+r+s+t+1\right)$ ,
9. $RAZI\left[CaTiO_{3}\right]=683.2696rst+666.80869\left(rs+rt+st+r+s+t+1\right)$ .

Proof. By Theorem 1, we have $M\left[CaTiO_{3}\left(r,s,t\right);x,y\right]=f\left(x,y\right)=12rstxy^{10}+3\left(r+1\right)\left(s+1\right)\left(t+1\right)x^{7}y^{10}+3\left(r+1\right)\left(s+1\right)\left(t+1\right)x^{7}y^{12}$ . Then

### 4.1 First zagreb index

$$\begin{array}{cc} f\left(x,y\right) & =12rstxy^{10}+3\left(r+1\right)\left(s+1\right)\left(t+1\right)x^{7}y^{10}+3\left(r+1\right)\left(s+1\right)\left(t+1\right)x^{7}y^{12},\\ D_{x}f\left(x,y\right) & =12rstxy^{10}+21\left(r+1\right)\left(s+1\right)\left(t+1\right)x^{7}y^{10}+21\left(r+1\right)\left(s+1\right)\left(t+1\right)x^{7}y^{12},\\ D_{y}f\left(x,y\right) & =120rstxy^{10}+30\left(r+1\right)\left(s+1\right)\left(t+1\right)x^{7}y^{10}+36\left(r+1\right)\left(s+1\right)\left(t+1\right)x^{7}y^{12},\\ \left(D_{x}+D_{y}\right)f\left(x,y\right) & =132rstxy^{10}+51\left(r+1\right)\left(s+1\right)\left(t+1\right)x^{7}y^{10}+57\left(r+1\right)\left(s+1\right)\left(t+1\right)x^{7}y^{12},\\ RM_{1}\left[CaTiO_{3}\right] & =\left(D_{x}+D_{y}\right)f\left(x,y\right){|}_{x=y=1}=240rst+108\left(rs+rt+st+r+s+t+1\right). \end{array}$$

### 4.2 Second zagreb index

$$\begin{array}{cc} f\left(x,y\right) & =12rstxy^{10}+3\left(r+1\right)\left(s+1\right)\left(t+1\right)x^{7}y^{10}+3\left(r+1\right)\left(s+1\right)\left(t+1\right)x^{7}y^{12},\\ D_{y}f\left(x,y\right) & =120rstxy^{10}+30\left(r+1\right)\left(s+1\right)\left(t+1\right)x^{7}y^{10}+36\left(r+1\right)\left(s+1\right)\left(t+1\right)x^{7}y^{12},\\ D_{x}D_{y}f\left(x,y\right) & =120rstxy^{10}+210\left(r+1\right)\left(s+1\right)\left(t+1\right)x^{7}y^{10}+252\left(r+1\right)\left(s+1\right)\left(t+1\right)x^{7}y^{12},\\ RM_{2}\left[CaTiO_{3}\right] & =D_{x}D_{y}f\left(x,y\right){|}_{x=y=1}=582rst+462\left(rs+rt+st+r+s+t+1\right). \end{array}$$

### 4.3 Modified second zagreb index

$$\begin{array}{cc} f\left(x,y\right) & =12rstxy^{10}+3\left(r+1\right)\left(s+1\right)\left(t+1\right)x^{7}y^{10}+3\left(r+1\right)\left(s+1\right)\left(t+1\right)x^{7}y^{12},\\ S_{y}f\left(x,y\right) & =\frac{12}{10}rstxy^{10}+\frac{3}{10}\left(r+1\right)\left(s+1\right)\left(t+1\right)x^{7}y^{10}+\frac{3}{12}\left(r+1\right)\left(s+1\right)\left(t+1\right)x^{7}y^{12},\\ S_{x}S_{y}f\left(x,y\right) & =\frac{12}{10}rstxy^{10}+\frac{3}{70}\left(r+1\right)\left(s+1\right)\left(t+1\right)x^{7}y^{10}+\frac{3}{84}\left(r+1\right)\left(s+1\right)\left(t+1\right)x^{7}y^{12},\\ R^{m}M_{2}\left[CaTiO_{3}\right] & =S_{x}S_{y}f\left(x,y\right){|}_{x=y=1}=1.27857rst+0.07857\left(rs+rt+st+r+s+t+1\right). \end{array}$$

### 4.4 General randic index

$$\begin{array}{cc} f\left(x,y\right) & =12rstxy^{10}+3\left(r+1\right)\left(s+1\right)\left(t+1\right)x^{7}y^{10}+3\left(r+1\right)\left(s+1\right)\left(t+1\right)x^{7}y^{12},\\ D_{y}^{\alpha }f\left(x,y\right) & =10^{\alpha }\left(12rstxy^{10}\right)+10^{\alpha }\left(3\left(r+1\right)\left(s+1\right)\left(t+1\right)x^{7}y^{10}\right)\\ & +12^{\alpha }\left(3\left(r+1\right)\left(s+1\right)\left(t+1\right)x^{7}y^{12}\right),\\ D_{x}^{\alpha }D_{y}^{\alpha }f\left(x,y\right) & =10^{\alpha }\left(12rstxy^{10}\right)+7^{\alpha }10^{\alpha }\left(3\left(r+1\right)\left(s+1\right)\left(t+1\right)x^{7}y^{10}\right)\\ & +7^{\alpha }12^{\alpha }\left(3\left(r+1\right)\left(s+1\right)\left(t+1\right)x^{7}y^{12}\right),\\ RR_{\alpha }\left[CaTiO_{3}\right] & =D_{x}^{\alpha }D_{y}^{\alpha }f\left(x,y\right){|}_{x=y=1}=\left(12\times 10^{\alpha }+3\times 7^{\alpha }\left(10^{\alpha }+12^{\alpha }\right)\right)rst\\ & +\left(3\times 7^{\alpha }\left(10^{\alpha }+12^{\alpha }\right)\right)\left(rs+rt+st+r+s+t+1\right). \end{array}$$

### 4.5 Forgotten index

$$\begin{array}{cc} f\left(x,y\right) & =12rstxy^{10}+3\left(r+1\right)\left(s+1\right)\left(t+1\right)x^{7}y^{10}+3\left(r+1\right)\left(s+1\right)\left(t+1\right)x^{7}y^{12},\\ D_{x}^{2}f\left(x,y\right) & =12rstxy^{10}+147\left(r+1\right)\left(s+1\right)\left(t+1\right)x^{7}y^{10}+147\left(r+1\right)\left(s+1\right)\left(t+1\right)x^{7}y^{12},\\ D_{y}^{2}f\left(x,y\right) & =1200rstxy^{10}+300\left(r+1\right)\left(s+1\right)\left(t+1\right)x^{7}y^{10}+432\left(r+1\right)\left(s+1\right)\left(t+1\right)x^{7}y^{12},\\ \left(D_{x}^{2}+D_{y}^{2}\right)f\left(x,y\right) & =1212rstxy^{10}+447\left(r+1\right)\left(s+1\right)\left(t+1\right)x^{7}y^{10}+579\left(r+1\right)\left(s+1\right)\left(t+1\right)x^{7}y^{12},\\ RFI\left[CaTiO_{3}\right] & =\left(D_{x}^{2}+D_{y}^{2}\right)f\left(x,y\right){|}_{x=y=1}=2238rst+1026\left(rs+rt+st+r+s+t+1\right). \end{array}$$

### 4.6 Harmonic index

$$\begin{array}{cc} f\left(x,y\right) & =12rstxy^{10}+3\left(r+1\right)\left(s+1\right)\left(t+1\right)x^{7}y^{10}+3\left(r+1\right)\left(s+1\right)\left(t+1\right)x^{7}y^{12},\\ Jf\left(x,y\right) & =12rstx^{11}+3\left(r+1\right)\left(s+1\right)\left(t+1\right)x^{17}+3\left(r+1\right)\left(s+1\right)\left(t+1\right)x^{19},\\ S_{x}Jf\left(x,y\right) & =\frac{12}{11}rstx^{11}+\frac{3}{17}\left(r+1\right)\left(s+1\right)\left(t+1\right)x^{17}+\frac{3}{19}\left(r+1\right)\left(s+1\right)\left(t+1\right)x^{19},\\ 2S_{x}Jf\left(x,y\right) & =\frac{24}{11}rstx^{11}+\frac{6}{17}\left(r+1\right)\left(s+1\right)\left(t+1\right)x^{17}+\frac{6}{19}\left(r+1\right)\left(s+1\right)\left(t+1\right)x^{19},\\ RH\left[CaTiO_{3}\right] & =2S_{x}Jf\left(x,y\right)f\left(x,y\right){|}_{x=1}=2.85055rst+0.66873\left(rs+rt+st+r+s+t+1\right). \end{array}$$

### 4.7 Symmetric degree division index

$$\begin{array}{cc} f\left(x,y\right) & =12rstxy^{10}+3\left(r+1\right)\left(s+1\right)\left(t+1\right)x^{7}y^{10}+3\left(r+1\right)\left(s+1\right)\left(t+1\right)x^{7}y^{12},\\ S_{y}f\left(x,y\right) & =\frac{12}{10}rstxy^{10}+\frac{3}{10}\left(r+1\right)\left(s+1\right)\left(t+1\right)x^{7}y^{10}+\frac{3}{12}\left(r+1\right)\left(s+1\right)\left(t+1\right)x^{7}y^{12},\\ D_{x}S_{y}f\left(x,y\right) & =\frac{12}{10}rstxy^{10}+\frac{21}{10}\left(r+1\right)\left(s+1\right)\left(t+1\right)x^{7}y^{10}+\frac{21}{12}\left(r+1\right)\left(s+1\right)\left(t+1\right)x^{7}y^{12},\\ D_{y}f\left(x,y\right) & =120rstxy^{10}+30\left(r+1\right)\left(s+1\right)\left(t+1\right)x^{7}y^{10}+36\left(r+1\right)\left(s+1\right)\left(t+1\right)x^{7}y^{12},\\ S_{x}D_{y}f\left(x,y\right) & =120rstxy^{10}+\frac{30}{7}\left(r+1\right)\left(s+1\right)\left(t+1\right)x^{7}y^{10}+\frac{36}{7}\left(r+1\right)\left(s+1\right)\left(t+1\right)x^{7}y^{12},\\ \left(D_{x}S_{y}+S_{x}D_{y}\right)f\left(x,y\right) & =\frac{606}{5}rstxy^{10}+\frac{447}{70}\left(r+1\right)\left(s+1\right)\left(t+1\right)x^{7}y^{10}+\frac{579}{84}\left(r+1\right)\left(s+1\right)\left(t+1\right)x^{7}y^{12},\\ RSDD\left[CaTiO_{3}\right] & =\left(D_{x}S_{y}+S_{x}D_{y}\right)f\left(x,y\right){|}_{x=y=1}=134.47857rst\\ & +13.27857\left(rs+rt+st+r+s+t+1\right). \end{array}$$

### 4.8 Inverse sum indeg index

$$\begin{array}{cc} f\left(x,y\right) & =12rstxy^{10}+3\left(r+1\right)\left(s+1\right)\left(t+1\right)x^{7}y^{10}+3\left(r+1\right)\left(s+1\right)\left(t+1\right)x^{7}y^{12},\\ D_{x}D_{y}f\left(x,y\right) & =120rstxy^{10}+210\left(r+1\right)\left(s+1\right)\left(t+1\right)x^{7}y^{10}+252\left(r+1\right)\left(s+1\right)\left(t+1\right)x^{7}y^{12},\\ JD_{x}D_{y}f\left(x,y\right) & =120rstx^{11}+210\left(r+1\right)\left(s+1\right)\left(t+1\right)x^{17}+252\left(r+1\right)\left(s+1\right)\left(t+1\right)x^{19},\\ S_{x}JD_{x}D_{y}f\left(x,y\right) & =\frac{120}{11}rstx^{11}+\frac{210}{17}\left(r+1\right)\left(s+1\right)\left(t+1\right)x^{17}+\frac{252}{19}\left(r+1\right)\left(s+1\right)\left(t+1\right)x^{19},\\ RISI\left[CaTiO_{3}\right] & =S_{x}JD_{x}D_{y}f\left(x,y\right){|}_{x=1}=36.52518rst+25.61609\left(rs+rt+st+r+s+t+1\right). \end{array}$$

### 4.9 Augmented zagreb index

$$\begin{array}{cc} f\left(x,y\right) & =12rstxy^{10}+3\left(r+1\right)\left(s+1\right)\left(t+1\right)x^{7}y^{10}+3\left(r+1\right)\left(s+1\right)\left(t+1\right)x^{7}y^{12},\\ D_{y}^{3}f\left(x,y\right) & =12000rstxy^{10}+3000\left(r+1\right)\left(s+1\right)\left(t+1\right)x^{7}y^{10}+5184\left(r+1\right)\left(s+1\right)\left(t+1\right)x^{7}y^{12},\\ D_{x}^{3}D_{y}^{3}f\left(x,y\right) & =12000rstxy^{10}+1029000\left(r+1\right)\left(s+1\right)\left(t+1\right)x^{7}y^{10}\\ & +1778112\left(r+1\right)\left(s+1\right)\left(t+1\right)x^{7}y^{12},\\ JD_{x}^{3}D_{y}^{3}f\left(x,y\right) & =12000rstx^{11}+1029000\left(r+1\right)\left(s+1\right)\left(t+1\right)x^{17}\\ & +1778112\left(r+1\right)\left(s+1\right)\left(t+1\right)x^{19},\\ Q_{-2}JD_{x}^{3}D_{y}^{3}f\left(x,y\right) & =12000rstx^{9}+1029000\left(r+1\right)\left(s+1\right)\left(t+1\right)x^{15}\\ & +1778112\left(r+1\right)\left(s+1\right)\left(t+1\right)x^{17},\\ S_{x}^{3}Q_{-2}JD_{x}^{3}D_{y}^{3}f\left(x,y\right) & =\frac{12000}{729}rstx^{9}+\frac{1029000}{3375}\left(r+1\right)\left(s+1\right)\left(t+1\right)x^{15}\\ & +\frac{1778112}{4913}\left(r+1\right)\left(s+1\right)\left(t+1\right)x^{17},\\ RAZI\left[CaTiO_{3}\right] & =S_{x}^{3}Q_{-2}JD_{x}^{3}D_{y}^{3}f\left(x,y\right){|}_{x=1}=683.2696rst\\ & +666.80869\left(rs+rt+st+r+s+t+1\right). \end{array}$$

## 5 Reverse degree and M-Polynomial based multifractal analysis of $CaTiO_{3}$ perovskite crystal

Multifractal Analysis is a specialized area within the dimension theory focused on examining the complexity of sets characterized by invariant local quantities, including Hurst exponents, Hausdorff dimensions, Holder exponents, and correlation dimensions. It serves as a valuable analytical tool for studying nonlinear systems (Brown et al., 1992). Multifractals can be viewed as an extension of fractals. A multifractal object exhibits greater complexity, remaining invariant under translation, while the scaling factor required to discern details varies based on the specific features being observed. A distinctive feature of multifractal analysis is its capacity to capture the self-similarities in the spatial arrangement of textons, essential microstructures in natural images, and information about complex structures. Multifractals have been proven that it is valuable in describing fluid and plasma turbulence, various material structures, crystal formations, and graphics (Mandelbrot, 1982; Saha et al., 2020; Lopes and Betrouni, 2009).

Through multifractal analysis, complex systems that are irregular in shape, amorphous, inhomogeneous, and anisotropic are characterized using geometric and statistical functions. Zinc oxide (ZnO) has been extensively investigated by researchers due to its potential applications in many devices, including electronic devices, pigments, catalysts and UV absorbers. Also, ZnO nanoparticles have been analyzed by the multifractal method. Significant applications, such as systematic categorization of crystal structures and studies on metastable crystallization, have recognized the importance of information-based assessments of structural intricacy (Saha et al., 2020; Lopes and Betrouni, 2009; Dafonte et al., 2015; Wang et al., 2012). This study utilizes a multifractal approach to gain insights into the intricate structures of complex crystals, a method that enables a deeper understanding of the unique characteristics and behaviors of these materials.

Renyi entropy is especially well-suited for capturing the multifractal characteristics of $CaTiO_{3}$ crystal geometry, as various structural domains can display differing levels of symmetry, compression and disorder. In $CaTiO_{3}$ contexts, it characterizes how generalized fractal dimensions are measured by Renyi entropy with spatial resolution, effectively capturing how the complexity of structure evolves or changes across different scales. Consequently, the use of Renyi entropy in GFD analysis provides a mathematically grounded and practically flexible method for measuring the structure of $CaTiO_{3}$ perovskite. To offer a deeper understanding of the hierarchical organization and inherent non-uniformity in $CaTiO_{3}$ perovskite crystal systems, it integrates concepts from information theory with spatial geometric analysis.

In this section, by using Equation 6, we calculate generalized fractal dimensions based on some reverse degree indices via M-polynomial. The complete general equations for the GFD of each reverse index are written as statements in Theorem 2. GFD spectra derived from the proposed methodology are used to understand the relative stability of the structures formed at each iteration of the $CaTiO_{3}$ crystal and the phase transition between each iteration. Let us now summarize the data presented in Tables 3, 4, which exhibit the values resulting from the generalized fractal dimensions Equation 6 based on the reverse degree and M-polynomial. The GFD values obtained for the $CaTiO_{3}$ perovskite crystal structure show distinct trends that vary based on the order q and the number of iterations where r=s=t . A general observation is that GFD values tend to decrease as the order q increases, across each layer iteration (where r=s=t ) of the topological indices $RM_{1}$ , $RM_{2}$ , $R^{m}M_{2}$ , $RR_{\alpha =2}$ , RFI , RH , RSDD , RISI , and RAZI .

**TABLE 3:** $D_{q}^{RTI}\left(M\left[CaTiO_{3}\left(r,s,t\right);x,y\right]\right)$ for $RM_{1}$ , $RM_{2}$ , $R^{m}M_{2}$ , $RR_{\alpha =2}\;\left(r=s=t\right)$ .

Indices	Layers	q = 2	q = 3	q = 4	q = 5	q = 6	q = 7	q = 8	q = 9	q = 10
$RM_{1}\left[CaTiO_{3}\right]$	r = s = t = 2	10.212	10.117	10.031	9.9543	9.886	9.8254	9.7715	9.7236	9.6809
	r = s = t = 3	**10.406**	**10.396**	**10.386**	**10.376**	**10.367**	**10.358**	**10.349**	**10.34**	**10.332**
	r = s = t = 4	10.298	10.233	10.17	10.108	10.049	9.992	9.9382	9.8875	9.84
	r = s = t = 5	10.137	9.9912	9.8506	9.7182	9.596	9.4853	9.3862	9.2982	9.2206
	r = s = t = 6	9.9829	9.7632	9.557	9.3701	9.2053	9.0627	8.9407	8.8368	8.7485
	r = s = t = 7	9.8487	9.5676	9.3108	9.0858	8.8943	8.7341	8.6012	8.491	8.3994
	r = s = t = 8	9.7343	9.4035	9.1082	8.8565	8.6481	8.478	8.3398	8.2272	8.1348
$RM_{2}\left[CaTiO_{3}\right]$	r = s = t = 2	7.7972	7.5097	7.3629	7.2692	7.1997	7.1434	7.0955	7.0536	7.0162
	r = s = t = 3	8.2511	7.9186	7.7379	7.6227	7.5394	7.4738	7.4192	7.3722	7.3308
	r = s = t = 4	8.5435	8.1968	7.998	7.8695	7.777	7.7049	7.6456	7.5951	7.551
	r = s = t = 5	8.7428	8.3937	8.1853	8.0483	7.9495	7.8729	7.8102	7.7571	7.7111
	r = s = t = 6	8.8856	8.539	8.3254	8.1828	8.0796	7.9996	7.9345	7.8795	7.8319
	r = s = t = 7	8.9923	8.6499	8.4336	8.2873	8.1809	8.0984	8.0313	7.9748	7.9261
	r = s = t = 8	**9.0748**	**8.7371**	**8.5194**	**8.3706**	**8.2617**	**8.1773**	**8.1087**	**8.0511**	**8.0014**
$R^{m}M_{2}\left[CaTiO_{3}\right]$	r = s = t = 2	**3.5602**	**2.8303**	**2.5265**	**2.3694**	**2.2747**	**2.2115**	**2.1664**	**2.1325**	**2.1062**
	r = s = t = 3	2.625	2.0502	1.8262	1.7123	1.6438	1.5981	1.5655	1.541	1.522
	r = s = t = 4	2.2072	1.7113	1.5233	1.4282	1.3711	1.333	1.3058	1.2854	1.2695
	r = s = t = 5	1.9743	1.5248	1.3569	1.2721	1.2212	1.1873	1.1631	1.1449	1.1308
	r = s = t = 6	1.8267	1.4074	1.2522	1.1739	1.127	1.0957	1.0733	1.0566	1.0435
	r = s = t = 7	1.7251	1.3269	1.1805	1.1067	1.0624	1.0329	1.0119	0.99604	0.98375
	r = s = t = 8	1.651	1.2684	1.1283	1.0579	1.0155	0.98733	0.96718	0.95207	0.94031
$RR_{\alpha =2}\left[CaTiO_{3}\right]$	r = s = t = 2	6.4605	6.2775	6.1369	6.0183	5.9169	5.83	5.7559	5.6925	5.6383
	r = s = t = 3	6.5375	6.3366	6.1895	6.0676	5.9641	5.876	5.8009	5.7368	5.682
	r = s = t = 4	6.5921	6.3788	6.2271	6.1028	5.9979	5.9089	5.8331	5.7685	5.7133
	r = s = t = 5	6.6322	6.41	6.2548	6.1288	6.0229	5.9331	5.8569	5.7919	5.7365
	r = s = t = 6	6.6627	6.4337	6.276	6.1487	6.042	5.9517	5.875	5.8098	5.7541
	r = s = t = 7	6.6866	6.4524	6.2926	6.1643	6.057	5.9662	5.8893	5.8238	5.768
	r = s = t = 8	**6.7058**	**6.4675**	**6.306**	**6.1769**	**6.069**	**5.978**	**5.9008**	**5.8351**	**5.7792**

The bolded values are representing the highest and the lowest topological indices at each order/category.

**TABLE 4:** $D_{q}^{RTI}\left(M\left[CaTiO_{3}\left(r,s,t\right);x,y\right]\right)$ for RFI , RH , RSDD , RISI , RAZI(r=s=t) .

Indices	Layers	q = 2	q = 3	q = 4	q = 5	q = 6	q = 7	q = 8	q = 9	q = 10
$RFI\left[CaTiO_{3}\right]$	r = s = t = 2	10.07	9.9032	9.7485	9.6081	9.4826	9.3715	9.274	9.1885	9.1137
	r = s = t = 3	**10.323**	**10.272**	**10.223**	**10.175**	**10.13**	**10.087**	**10.046**	**10.007**	**9.9707**
	r = s = t = 4	10.256	10.181	10.114	10.053	9.999	9.9511	9.9085	9.8708	9.8372
	r = s = t = 5	10.123	9.9862	9.8615	9.7493	9.6492	9.5604	9.4817	9.4121	9.3503
	r = s = t = 6	9.9887	9.7891	9.6087	9.4488	9.3091	9.188	9.0835	8.9934	8.9155
	r = s = t = 7	9.8683	9.6145	9.3882	9.1918	9.0242	8.8826	8.7633	8.6628	8.5777
	r = s = t = 8	9.7643	9.4652	9.2026	8.9791	8.7925	8.638	8.5105	8.4047	8.3166
$RH\left[CaTiO_{3}\right]$	r = s = t = 2	**9.4041**	**8.9424**	**8.5573**	**8.2516**	**8.0145**	**7.8314**	**7.6889**	**7.5765**	**7.4866**
	r = s = t = 3	8.1375	7.3367	6.8008	6.4484	6.2109	6.0444	5.9229	5.831	5.7592
	r = s = t = 4	7.3371	6.4293	5.886	5.5545	5.3407	5.1944	5.0889	5.0095	4.9477
	r = s = t = 5	6.819	5.8767	5.3474	5.0364	4.8397	4.7063	4.6104	4.5385	4.4824
	r = s = t = 6	6.4626	5.51	4.9963	4.7009	4.5161	4.3913	4.3018	4.2346	4.1823
	r = s = t = 7	6.2042	5.2505	4.7502	4.4668	4.2905	4.1717	4.0867	4.0228	3.9732
	r = s = t = 8	6.0089	5.0576	4.5686	4.2943	4.1244	4.0102	3.9284	3.8671	3.8193
$RSDD\left[CaTiO_{3}\right]$	r = s = t = 2	**5.3441**	**4.4196**	**3.9743**	**3.7318**	**3.5834**	**3.484**	**3.4129**	**3.3596**	**3.3181**
	r = s = t = 3	4.0673	3.266	2.9198	2.7389	2.6295	2.5565	2.5043	2.4652	2.4347
	r = s = t = 4	3.4657	2.749	2.453	2.3004	2.2085	2.1471	2.1033	2.0705	2.0449
	r = s = t = 5	3.1224	2.4603	2.1936	2.057	1.9747	1.9199	1.8807	1.8513	1.8284
	r = s = t = 6	2.9019	2.2773	2.0294	1.9029	1.8268	1.7761	1.7398	1.7127	1.6915
	r = s = t = 7	2.749	2.1512	1.9165	1.797	1.7251	1.6772	1.643	1.6173	1.5973
	r = s = t = 8	2.6368	2.0593	1.8342	1.7198	1.651	1.6052	1.5724	1.5478	1.5287
$RISI\left[CaTiO_{3}\right]$	r = s = t = 2	8.5254	8.2149	8.0524	7.9577	7.8964	7.8531	7.8206	7.7947	7.7734
	r = s = t = 3	9.1103	8.8048	8.6198	8.5023	8.423	8.3663	8.3236	8.29	8.2627
	r = s = t = 4	9.45	9.1769	8.9948	8.8708	8.7833	8.7192	8.6703	8.6317	8.6002
	r = s = t = 5	9.6618	9.4223	9.2515	9.1288	9.039	8.9713	8.9189	8.8772	8.843
	r = s = t = 6	9.8027	9.5919	9.4342	9.3163	9.2271	9.1583	9.1042	9.0606	9.0248
	r = s = t = 7	9.9014	9.7142	9.569	9.457	9.3699	9.3015	9.2468	9.2022	9.1653
	r = s = t = 8	**9.9736**	**9.8054**	**9.6715**	**9.5654**	**9.4813**	**9.4139**	**9.3593**	**9.3143**	**9.2768**
$RAZI\left[CaTiO_{3}\right]$	r = s = t = 2	6.647	6.5795	6.535	6.4972	6.4628	6.4309	6.401	6.3729	6.3466
	r = s = t = 3	6.7043	6.6233	6.5739	6.5336	6.4978	6.4649	6.4343	6.4058	6.379
	r = s = t = 4	6.745	6.6546	6.6017	6.5597	6.5229	6.4893	6.4582	6.4292	6.4022
	r = s = t = 5	6.7751	6.6777	6.6223	6.579	6.5414	6.5073	6.4758	6.4466	6.4194
	r = s = t = 6	6.7979	6.6953	6.638	6.5937	6.5555	6.521	6.4893	6.4598	6.4325
	r = s = t = 7	6.8158	6.7092	6.6503	6.6053	6.5666	6.5318	6.4999	6.4703	6.4428
	r = s = t = 8	**6.8303**	**6.7204**	**6.6603**	**6.6146**	**6.5756**	**6.5405**	**6.5084**	**6.4787**	**6.4511**

The bolded values are representing the highest and the lowest topological indices at each order/category.

Looking at Tables 3, 4, especially for topological indices such as $R^{m}M_{2}$ , RH , and RSDD , the GFD values consistently decline with increasing q and with the number of iterations (r=s=t) increases. In contrast, for certain other topological indices namely, $RM_{2}$ , $RR_{\alpha =2}$ , RISI , and RAZI , the GFD values show an increasing trend when the number of iterations (r=s=t) is raised, especially at q=2 . Moreover, for these same indices, GFD values also increase with each successive order of q , provided the iteration layers count is rising.

On the other hand, the $RM_{1}$ and RFI indices show a different behavior: their GFD values decrease as the order q increases, irrespective of the iteration level. When the iteration count is increased beyond r=s=t=3 , this downward trend becomes even more noticeable, as shown in Tables 3, 4, because the GFD values for these indices continuously decrease over all orders of q . Overall, GFD values of the $CaTiO_{3}$ perovskite crystal structure patterns show how sensitively the iteration depth and Renyi entropy order q depend, with different topological indices reacting differently under these circumstances, as illustrated in Tables 3, 4.

**FIGURE 2:**
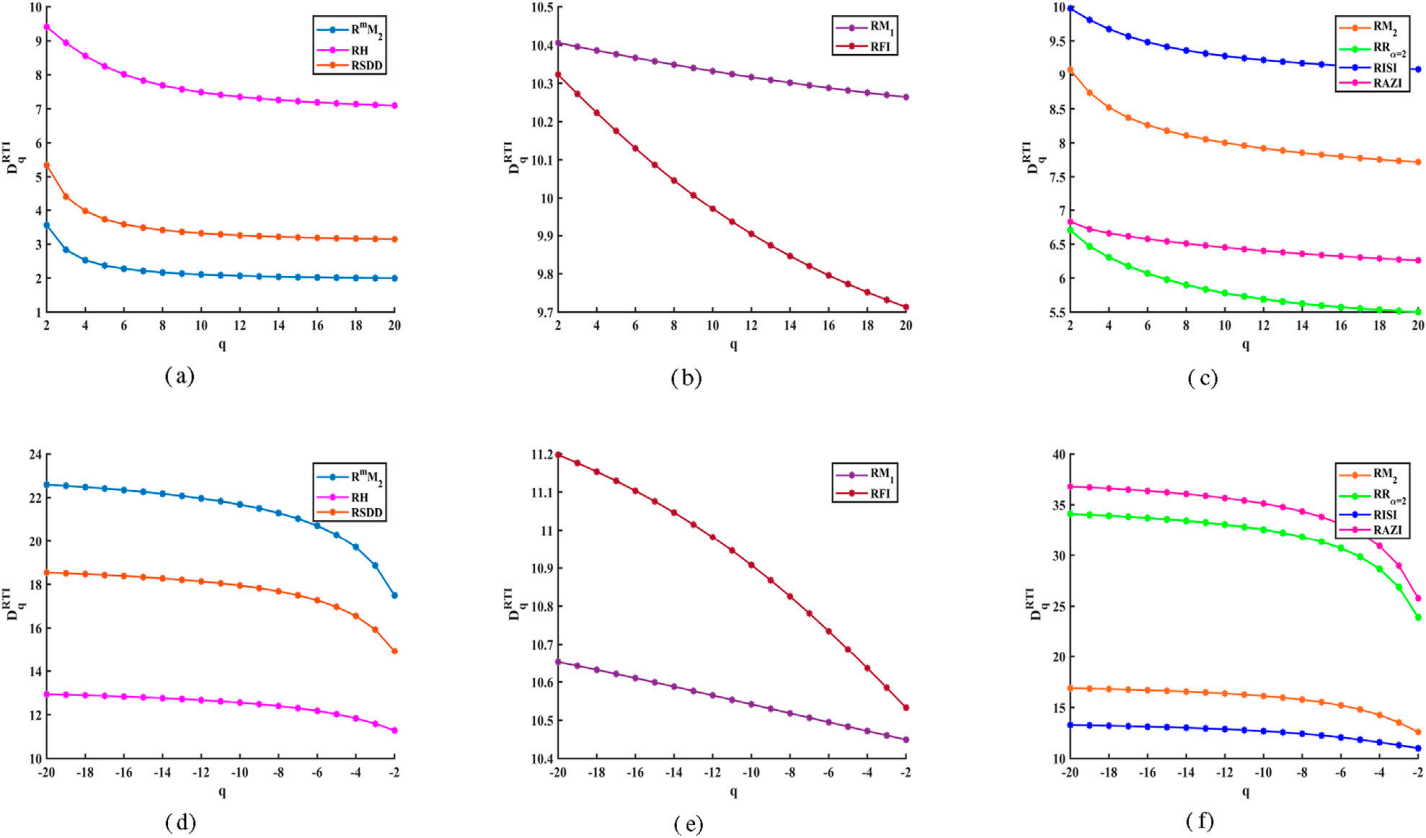
Reverse degree and M-polynomial based GFD values for $CaTiO_{3}$ crystal. **(a)** [r = s = t = 2]. **(b)** [r = s = t = 3]. **(c)** [r = s = t = 8]. **(d)** [r = s = t = 2]. **(e)** [r = s = t = 3]. **(f)** [r = s = t = 8].

The analysis of GFD using M-polynomial by reverse degree was conducted based on topological indices for $CaTiO_{3}$ crystal with detailed interpretations of results. Figure 2 presents a comparison of the reverse degree-based topological indices across different specific iterations of r=s=t . Additionally, Figure 2 illustrates the differences in GFD values derived from topological indices for both positive and negative values of order q . We then use structural complexity parameters based on GFD information in Tables 3, 4 to statistically evaluate. From Figures 2c–h, it can be observed that as the positive values of the order q increase, GFD values derived from the topological indices decrease. Conversely, when the negative values of q become smaller, GFD values increase. Specifically, the GFD curves based on the reverse degree via M-polynomial for the $CaTiO_{3}$ crystal structure, under increasing positive q , show a descending trend that can be ranked as follows in Figures 2a–c:

$$\begin{array}{cc} & •Atr=s=t=2:RH>RSDD>R^{m}M_{2}\\ & •Atr=s=t=3:RM_{1}>RFI\\ & •Atr=s=t=8:RISI>RM_{2}>RAZI>RR_{\alpha =2} \end{array}$$

Likewise, Figures 2d–f indicate that the GFD curves increase as the negative values of the order q become more negative. In a similar manner, when both the order q and the number of iterations where r=s=t are assigned more larger values, the behavior of topological indices remains consistent with the previously observed trends. That is, the general pattern in the variation of GFD values, whether for increasing positive or decreasing negative q , continues to follow the same qualitative nature, regardless of how high the values of q and the iteration layer parameters become. Furthermore, these topological indices imply a stable and predictable relationship, even when measured for higher levels of $CaTiO_{3}$ crystal.

The growth and transformation of crystalline phases are geological phenomena that occur both globally and locally, which can be analyzed through complexity measures using GFD of the $CaTiO_{3}$ crystal. GFD information based complexity measurements of $CaTiO_{3}$ can evaluate how readily the material crystallizes, guided by the principle of simplicity. This approach provides a powerful framework for studying the emergence and progression of both structurally simple and highly complex crystalline phases, whether occurring naturally within geological environments or synthesized under controlled laboratory experiments.

The use of modularity, a fundamental design principle in many inorganic crystal structures, has greatly aided the investigation of structural categorization, relationships, description, and structure prediction. Thus, the concept of maximal simplicity in the modular crystallographic structure of $CaTiO_{3}$ can be represented through GFD values; greater data storage and processing capabilities for more complex structures can be realized through GFD. The existing topological descriptors and GFD measures for various phases of $CaTiO_{3}$ -3D materials can be used to predict physicochemical aspects like potential energy, mechanical, and optical characteristics. In addition, properties such as atomic charges and molecular hardness offer a solid foundation for predicting molecular connectivity. The primary purpose of the data in Tables 3, 4 is to directly link them to the properties of the $CaTiO_{3}$ crystal through the correlation model in Section 6.

## 6 Correlation based predictive analysis on physical properties of $CaTiO_{3}$

The aim is to present GFD concept based on topological indices, investigate the relationship between these values and the chemical features of compounds, and utilize them in QSPR and QSAR analyzes. We will conduct a correlation analysis on the examined structures to identify key topological indices that most effectively predict physical properties. The Molecular Mass (MM) and Collision Diameter (CD) help to predict its behavior in various applications and to characterize the crystal. Molecular mass provides information about the total mass of the crystal, affecting the density, stability, and reactivity of a crystal during chemical reactions. In thermodynamic calculations, it also contributes to the determination of melting points and phase transitions. Understanding how substances travel through a material and how diffusion occurs inside the crystal structure depends on the collision diameter. Additionally, it clarifies how inter-molecular interactions affect characteristics like heat conductivity and mechanical strength. A limited set of physical and chemical properties of $CaTiO_{3}$ crystal, such as molecular mass and collision diameter, for different iterations of $CaTiO_{3}$ crystal material are listed in Table 5 using Chemcraft 1.8 software. In this section, we explore the relationship between the potential properties of the selected $CaTiO_{3}$ crystal noted in Table 5 and GFD values of topological indices from Tables 3, 4.

**TABLE 5:** Physical properties of $CaTiO_{3}\left(r,s,t\right)$ crystal.

Structure	Molecular Mass (g/mol)	Collision diameter (angstrom $\left(\overset{{}^{\circ}}{A}\right)$ )
$CaTiO_{3}\left(2,2,2\right)$	3341.7461	14.4644
$CaTiO_{3}\left(3,3,3\right)$	7986.4380	18.3539
$CaTiO_{3}\left(4,4,4\right)$	15748.8107	22.2434
$CaTiO_{3}\left(5,5,5\right)$	27444.2357	26.1328
$CaTiO_{3}\left(6,6,6\right)$	43888.0848	30.0223
$CaTiO_{3}\left(7,7,7\right)$	65895.7297	33.9118
$CaTiO_{3}\left(8,8,8\right)$	94282.5419	37.8012

As the iterations of the $CaTiO_{3}$ crystal structure increase, there is a corresponding rise in its physical properties, notably the molecular mass and collision diameter. This trend indicates that with each iteration that the overall size and mass of the $CaTiO_{3}$ crystals become greater, influencing their interactions and behaviors. The parameters listed in Table 5 will be valuable for establishing correlations and creating a plot for the linear fit analysis. We utilize distinctive crystal structures to determine the physical properties of samples such as $CaTiO_{3}\left(2,2,2\right)$ , $CaTiO_{3}\left(3,3,3\right)$ , $CaTiO_{3}\left(4,4,4\right)$ , $CaTiO_{3}\left(5,5,5\right)$ , $CaTiO_{3}\left(6,6,6\right)$ , $CaTiO_{3}\left(7,7,7\right)$ , and $CaTiO_{3}\left(8,8,8\right)$ .

Table 6 displays the strength of the relationship between GFD and physical properties of $CaTiO_{3}$ crystals for orders q=2 and 10, based on correlations across all indices. RAZI index shows the highest correlation coefficient value for molecular mass and collision diameter at q=2 and q=10 orders. RAZI index showing the strongest correlation with physical properties, is useful for predicting molecular behavior according to the framework. This strong correlation typically arises from a structural or mathematical basis, because the RAZI index captures properties like branching, connectivity, and molecular size that directly impact physical features. Their effectiveness depends on how accurately their mathematical formulation reflects the structural factors that determine the property. Consequently, when assessing the relationship between GFD and physical properties of $CaTiO_{3}$ crystals, GFD derived from RAZI index indicates a stronger correlation. Furthermore, compared to other indices, $R^{m}M_{2}$ and RFI indices exhibit the lowest correlation coefficient values for molecular mass and collision diameter at orders q=2 and q=10 . The $R^{m}M_{2}$ and RFI indices that have the lowest correlation coefficient values with physical properties are generally less effective at forecasting that particular property because they are unable to adequately capture the relevant structural features. Nonetheless, the $R^{m}M_{2}$ and RFI indices can still be useful for predicting other properties, and their weak correlations can help identify which structural characteristics have minimal influence on a specific property.

**TABLE 6:** Correlation coefficients between physical properties of $CaTiO_{3}$ and $D_{q}^{RTI}\left(M\left[CaTiO_{3}\left(r,s,t\right);x,y\right]\right)$ .

Indices	Molecular Mass (MM)		Collision diameter (CD)	
	q = 2	q = 10	q = 2	q = 10
$RM_{1}\left[CaTiO_{3}\right]$	−0.9474	−0.9296	−0.9062	−0.9180
$RM_{2}\left[CaTiO_{3}\right]$	0.8480	0.8711	0.9564	0.9692
$R^{m}M_{2}\left[CaTiO_{3}\right]$	**−0.7669**	**−0.7601**	−0.9022	−0.8971
$RR_{\alpha =2}\left[CaTiO_{3}\right]$	0.8744	0.8765	0.9709	0.9720
$RFI\left[CaTiO_{3}\right]$	−0.8901	−0.8537	**−0.8063**	**−0.7660**
$RH\left[CaTiO_{3}\right]$	−0.8335	−0.7789	−0.9480	−0.9108
$RSDD\left[CaTiO_{3}\right]$	−0.7778	−0.7647	−0.9102	−0.9006
$RISI\left[CaTiO_{3}\right]$	0.8150	0.8672	0.9362	0.9671
$RAZI\left[CaTiO_{3}\right]$	**0.8752**	**0.8766**	**0.9713**	**0.9721**

The bolded values are representing the highest and the lowest topological indices at each order/category.

A extensive correlation analysis of $CaTiO_{3}$ perovskite crystals demonstrated that certain topological indices are not appropriate for the chosen structure, as they showed weak correlation coefficients, indicating limited relevance or predictive capability in this instance. Such weakly correlated topological indices can still be effective for forecasting other properties or in different types of materials, which vary depending on the molecular structure. Since each physical property is affected by distinct structural characteristics, an index that shows a poor correlation with one property might exhibit a strong correlation with another. Moreover, these indices can offer meaningful insights when used alongside others in multivariate modeling approaches. Furthermore, even when the value of order q is changed, the correlation coefficient values for molecular mass and collision diameter properties obtained by correlating with GFD based on the reverse degree based topological indices via M-polynomial give similar results. This consistency indicates that the relationship between the topological descriptors and the physical properties of the $CaTiO_{3}$ structure is robust, even when the entropy parameter q varies.

**FIGURE 3:**
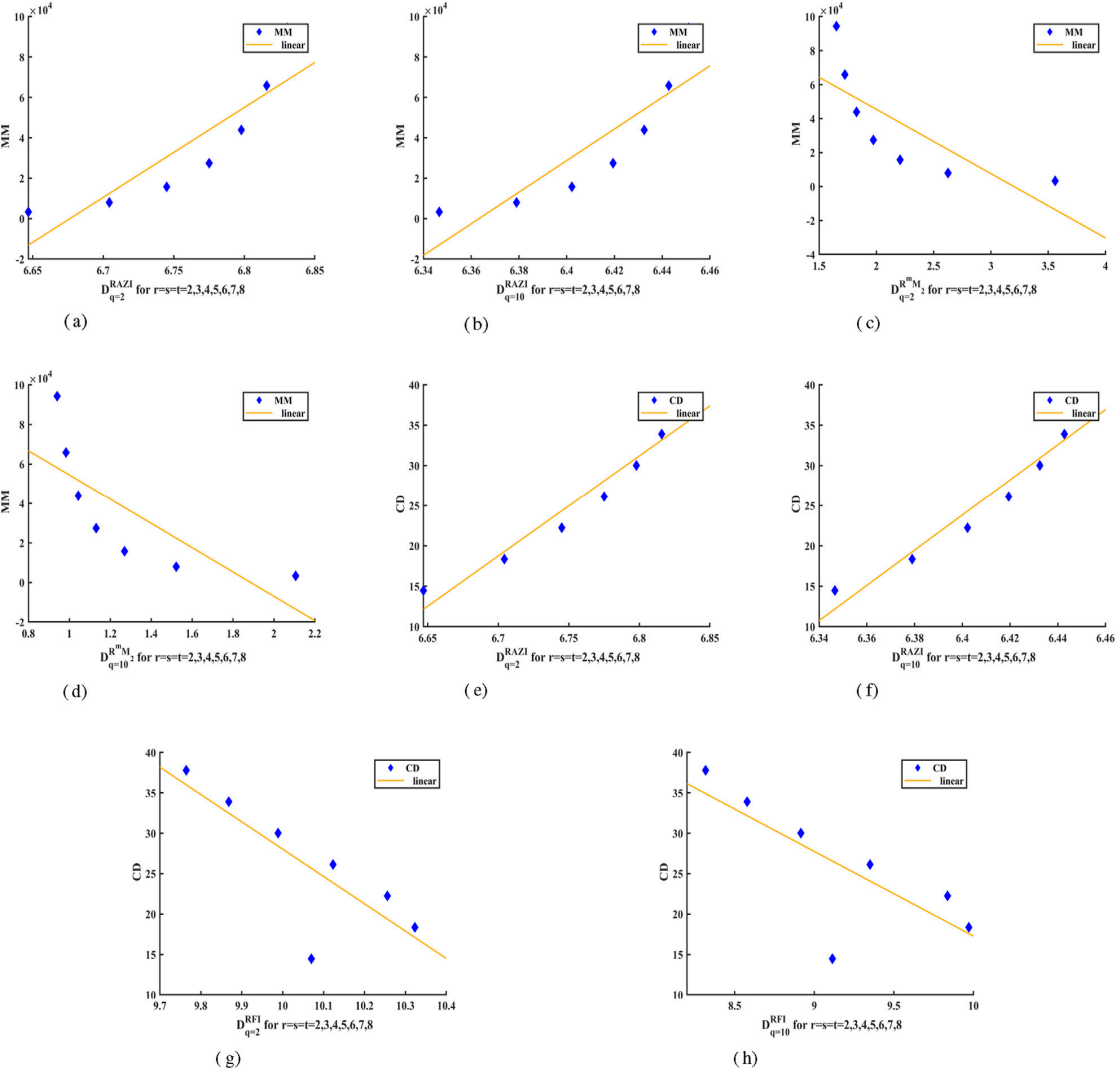
Linear Fitting of GFD values with Properties of $CaTiO_{3}$ Crystal. **(a)** [MM:RAZI(q=2)] . **(b)** [MM:RAZI(q=10)] . **(c)** $\left[MM:R^{m}M_{2}\left(q=2\right)\right]$ . **(d)** $\left[MM:R^{m}M_{2}\left(q=10\right)\right]$ . **(e)** [CD:RAZI(q=2)] . **(f)** [CD:RAZI(q=10)] . **(g)** [CD:RFI(q=2)] . **(h)** [CD:RFI(q=10)] .

Figure 3 presents a linear regression analysis derived from the characteristics of $CaTiO_{3}$ crystal, with particular emphasis on those topological indices that demonstrate either strong or weak correlation coefficients, as indicated by the data in Table 6. Figures 3a–h demonstrate how the structural characteristics of the crystal, such as molecular mass and collision diameter, are linked to its complexity as evaluated through generalized fractal dimensions. Furthermore, this study establishes a correlation between GFD values derived through different topological indices and the experimentally determined physical properties of the $CaTiO_{3}$ crystal, particularly its molecular mass and collision diameter. This integrated approach not only strengthens the use of GFD in measuring complexity of structure but also connects it with material properties capable of providing theoretical explanations.

## 7 Conclusion

In this context, the precise analytical equations are derived for topological descriptors using a graph-theoretic edge partitioning method, focusing on various reverse degree based topological indices through M-polynomial of the $CaTiO_{3}$ crystal. Then the proposed GFD is defined using reverse degree and M-polynomial based topological indices. Additionally, we utilized the equations obtained in that analysis to determine the GFD values for the $CaTiO_{3}$ crystal structure, presenting the results both in table format and graphically. Moreover, this approach offers a structure for differentiating various nanostructural configurations of $CaTiO_{3}$ using topological indices. This could have important implications for materials design, as slight variations in structure can have a major impact on functional properties. Additionally, we show that the GFD based descriptors correlate with established material attributes, indicating their potential usefulness for predictive modeling in molecular informatics. It is aimed primarily in this study to explore how these findings can assist in QSPR analyzes of perovskite crystals. We investigated their effectiveness in predicting specific physical properties of the $CaTiO_{3}$ crystal using linear fit analysis. A comprehensive correlation analysis revealed that several topological indices are suitable for the selected structures because they exhibit high correlation coefficients. Furthermore, the results of the correlation analysis were consistent for $RM_{2}$ , $RR_{\alpha =2}$ , RISI , and RAZI indices. Notably, molecular mass and collision diameter showed a strong correlation with GFD according to the RAZI index. $CaTiO_{3}$ crystal studies demonstrate the benefits for future studies of applying mathematical findings to various fields, namely, the design of electronic components such as capacitors and ferroelectric devices, to accurately predict their electrical properties.

## Data Availability

The original contributions presented in the study are included in the article/supplementary material, further inquiries can be directed to the corresponding author.
